# Supplementary material for: Efficient Photocatalytic Hydrogen Production over NiS-Modified Cadmium and Manganese Sulfide Solid Solutions
Source: Materials (Basel). 2022 Nov 14;15(22):8026. doi: 10.3390/ma15228026 (PMC9696279; doi:10.3390/ma15228026)
Supplement: Supplementary file 1 [file materials-15-08026-s001.zip › materials-2012269-supplementary.pdf]

Supporting Information

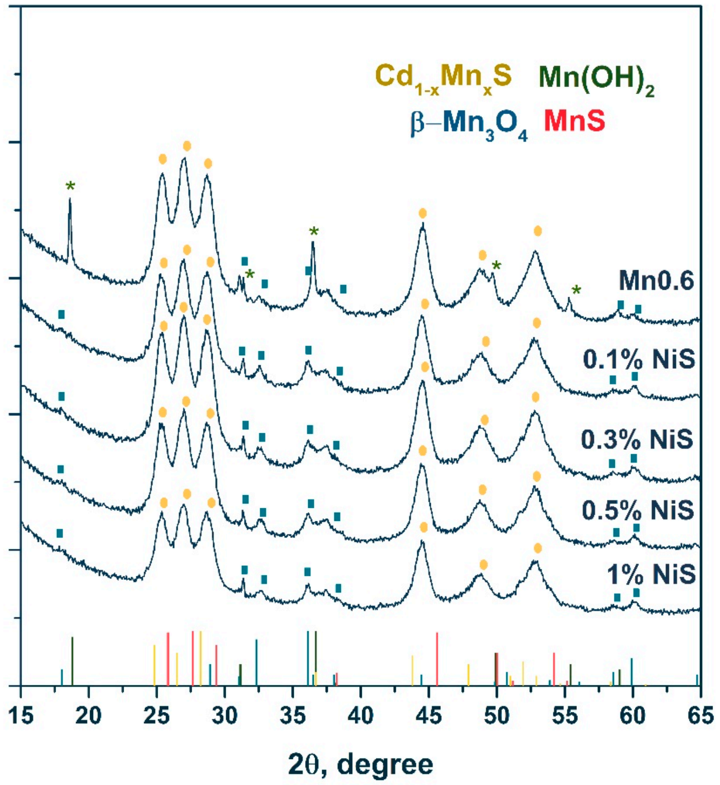

Figure S1. XRD patterns of the NiS/Mn0.6 HT120 samples.

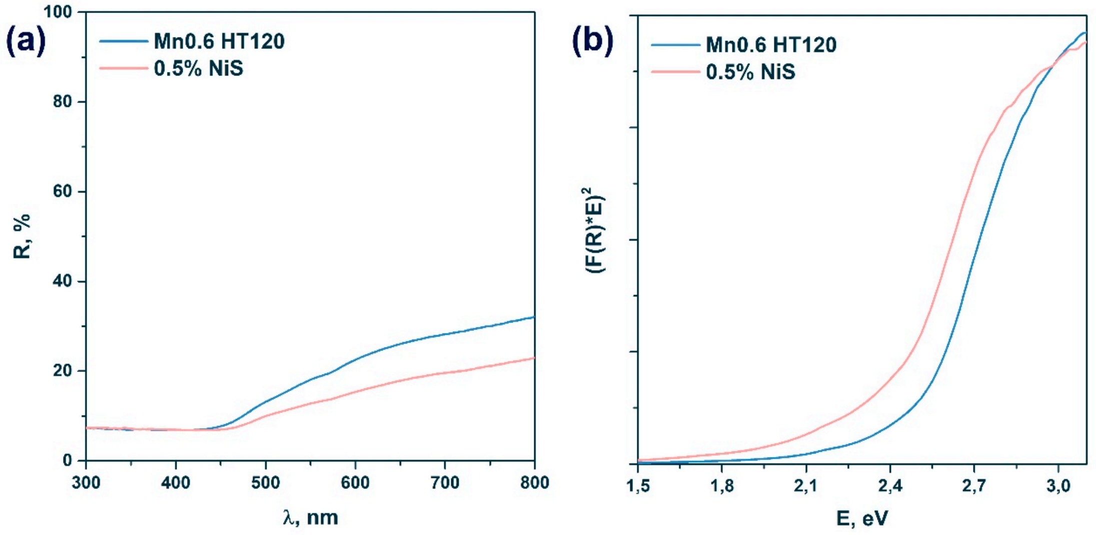

Figure S2. Diffuse reflectance spectra (**a**) and Tauc's plot (**b**) of the NiS/Mn0.6 HT120 samples

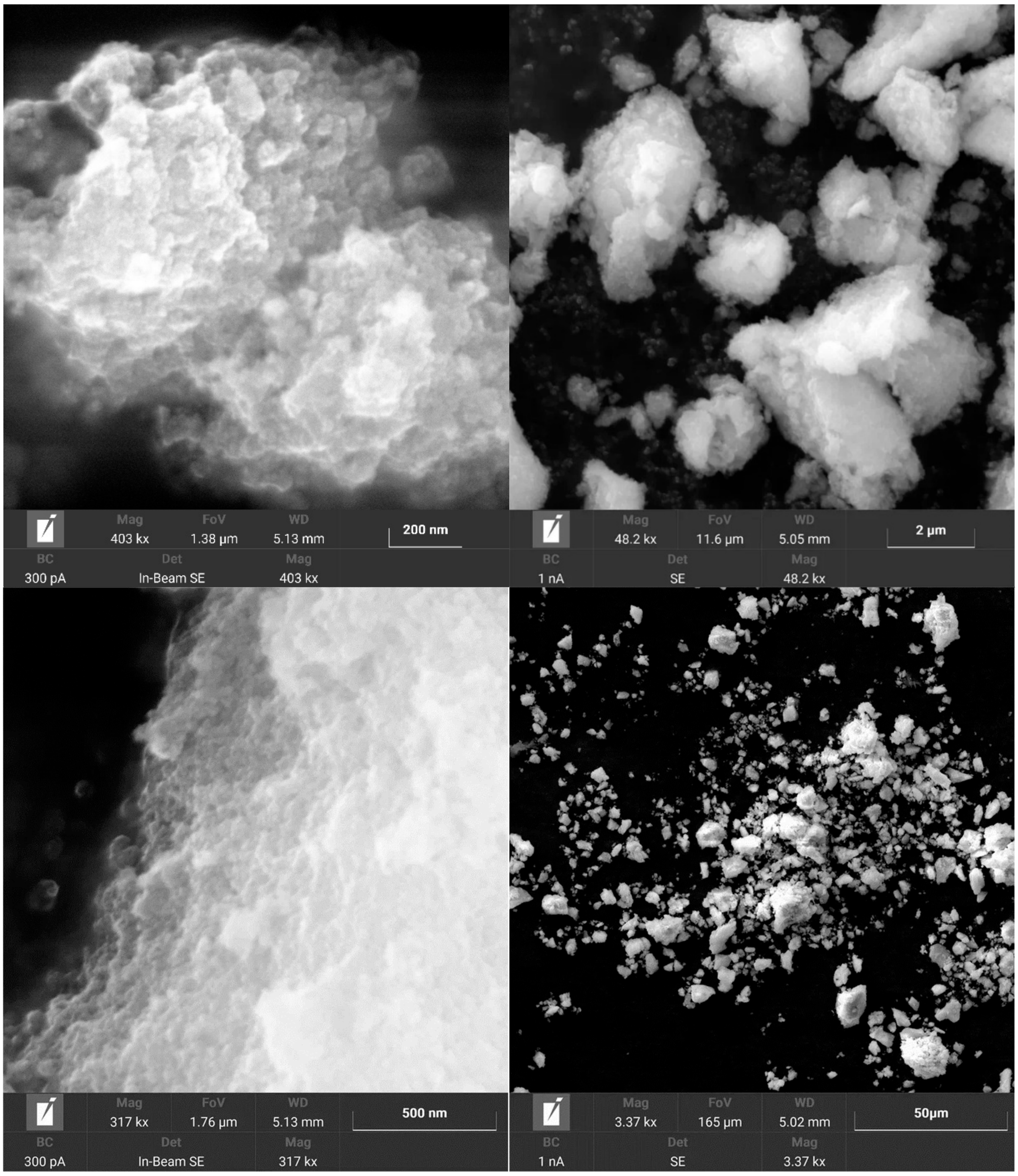

Figure S3. Focused ion beam FE-SEM images of 0.1% NiS/Mn0.6 HT120 photocatalyst.
